# Ethnic discrimination and health: the relationship between experienced ethnic discrimination and multiple health domains in Norway's rural Sami population

**DOI:** 10.3402/ijch.v74.25125

**Published:** 2015-02-13

**Authors:** Ketil Lenert Hansen

**Affiliations:** Centre for Sami Health Research, Institute of Community Medicine, UiT The Arctic University of Norway, Tromso, Norway

**Keywords:** ethnic discrimination, racism, ethnicity, Sami, indigenous, health, Norway

## Abstract

**Objective:**

Self-reported ethnic discrimination has been associated with a range of health outcomes. This study builds on previous efforts to investigate the prevalence of self-reported ethnic discrimination in the indigenous (Sami) population, and how such discrimination may be associated with key health indicators.

**Study design:**

The study relies on data from the 2003/2004 (n=4,389) population-based study of adults (aged 36–79 years) in 24 rural municipalities of Central and North Norway (the SAMINOR study). Self-reported ethnic discrimination was measured using the question: “Have you ever experienced discrimination due to your ethnic background?” Health indicators included questions regarding cardiovascular disease, diabetes, chronic muscle pain, metabolic syndrome and obesity. Logistic regression was applied to examine the relationship between self-reported ethnic discrimination and health outcomes.

**Results:**

The study finds that for Sami people living in minority areas, self-reported ethnic discrimination is associated with all the negative health indicators included in the study.

**Conclusion:**

We conclude that ethnic discrimination affects a wide range of health outcomes. Our findings highlight the importance of ensuring freedom from discrimination for the Sami people of Norway.

In recent years, evidence has been produced indicating that discrimination is an important determinant of health inequities (1). A growing body of research shows strong association between self-reported ethnic discrimination and poor health outcomes across diverse minority groups in different countries (2–7). Discrimination has been found to be associated with negative mental and physical health outcomes including cardiovascular disease (CVD) (8–10), diabetes (11,12), and obesity (13), and negative health behaviours (14) and increased mortality (5,6,7,15). Research into ethnic discrimination generally focuses on adult populations (16); adults belonging to indigenous minorities more frequently report having experienced ethnic discrimination. In Norway, Sami adults report experienced ethnic discrimination more frequently in comparison to ethnic Norwegians (17,18). Experienced ethnic discrimination is associated with adverse self-reported overall health status (19) and psychological stress (symptoms of anxiety and depression) (20).

The Sami are the indigenous people of *Sápmi*, a territory comprising parts of Arctic Norway, Sweden, Finland and Russia's Kola Peninsula. The Sami language belongs to the Finno-Ugric branch of the Uralic language family. The Sami are engaged in a variety of livelihoods, including farming, fishing, trapping and reindeer husbandry (breeding and herding). Traditional means of subsistence – continuing to this day – such as reindeer husbandry, often in combination with small-scale fishing and agriculture, forms the economic backbone of Sami communities (21). Today, the Sami are represented in practically all the modern professions and trades; a majority of the Sami population has adopted the Western lifestyle (modern professions; food habits). Only small groups are still holding on to traditional ways of life (based on fishing, hunting and reindeer herding) (22). The Sami people are estimated to comprise between 60,000 and 110,000 individuals residing in these 4 countries (21). Approximately 70% of the Sami population lives in the Norwegian part of Sápmi. In recent decades, there has been considerable migration from traditional Sami municipalities to urban areas, implying a significant Sami (or multi-ethnic) population living in Norwegian towns and cities (23). Nonetheless, this study has been specifically designed for Sami people living in rural communities with less than 3,000 inhabitants.

An overview of recent research reveals that the Sami people are in a uniquely positive situation in terms of health in comparison to other indigenous peoples of the circumpolar area (22). The Norwegian Sami have about the same life expectancies and mortality rates as the majority of population (24,25). The Sami are less exposed to many of the health issues (i.e. dramatically elevated risk of diabetes, CVD, lung cancer and various infectious diseases) frequently faced by indigenous populations of the circumpolar region and elsewhere. In general, the variance in risk of the major diseases and causes of death is minor (26). North Norway is widely known to have higher mortality due to CVD than South Norway (irrespective of ethnicity). In the first “Finnmark Study” (1974–1975), Sami/Finnish males aged 35–49 were shown to have an elevated cardiovascular risk of 40% (in comparison to ethnic Norwegians) (27). Presently, however, prevalence data and follow-ups have shown the variance in risk factors and risk of CVD to be negligible (28–30). In terms of mortality due to CVD, conflicting results have been presented (31,32). Various definitions of Sami ethnicity have been used in these studies (33). The prevalence of obesity in Sami women has been shown to be higher than for Ethnic Norwegian women (34); the apoB/apoA-1 ratios and cholesterol levels of middle-aged Sami people have been found to be somewhat higher than in non-Sami people (35). Higher prevalence of diabetes has been documented (35). Sami females have a significantly elevated risk of developing type 2 diabetes mellitus (36); Sami individuals living in majority areas show reduced prevalence of chronic muscle pain in comparison to individuals belonging to the majority population living in minority areas (37). Kvernmo and Eckhoff (38) found a strong association between musculoskeletal pain and anxiety/depression in indigenous and non-indigenous adolescents living in the Arctic.

Some of the health problems that the Sami people contend with originate in the colonization, discrimination, rapid modernization and marginalization of the Sami identity and culture (22). As a consequence of acculturation, related to the process of colonization and forced assimilation over centuries, the Sami people have been subject to discrimination and prejudice, as have indigenous peoples in other parts of the world (17,18,39).

The assimilation policies of the Norwegian government date back to around 1850. The Sami people were coerced into adopting the Norwegian language and identity. The process – known as “Norwegianization” – fundamentally altered the value structure of Sami culture (40). This was particularly evident in educational institutions; the Sami language was banned and Sami children were sent to boarding schools in order to remove them from their linguistic and cultural environment (41,42). The Sami were subject to hostile attitudes (discrimination) that practically permeated society, causing many Sami persons to lose their ethnic identity, culture, language and traditional knowledge – particularly in Sami minority areas, in which assimilation efforts were more intense (40). The Sami culture and identity have been affected by the development of the Norwegian nation-state since World War II; people increasingly abandoned the primary industries in favour of secondary and tertiary industries as the nation recovered. Regional administrative centres (traditionally Norwegian-dominated) gained importance, while Sami communities were drained of employment opportunities and resources. In all, this affected Sami culture and identity negatively (43). Despite the recent revival and revitalization of Sami identity, culture and language in Norwegian society, the Sami report high rates of ethnic discrimination (17,39). The protective (“buffer”) effect of a stronger Sami civil society is more apparent in Sami majority areas (in which the Sami population is more proficient in the Sami language and culture, Sami institutions are well-established and include professional indigenous health and social services) (19,21).

“Ethnicity” is the description of an individual based on race or culture of origin. Thus, ethnic discrimination refers to unfair treatment due to one's ethnicity (44). Ethnic discrimination can be communicated explicitly or implicitly, and occurs at 3 levels: internalized (i.e. the incorporation of racist attitudes, beliefs or ideologies), interpersonal (interaction between individuals) and structural (i.e. institutional policies that restrict access to opportunities or resources). Ethnic discrimination may be communicated in a number of ways (e.g. intentionally, subtly, unwittingly or unconsciously) (3). Ethnic discrimination may be articulated through beliefs (e.g. prejudice and negative stereotypes), emotions (e.g. fear/hatred) or behaviours/practices (e.g. unfair treatment) ranging from open threats and insults (including physical violence) to phenomena deeply embedded in social systems and structures (16). All forms of discrimination can have individual as well as population level effects.

Ethnic discrimination has a number of implications in regards to health (6,44), but they are not easily measured. The debate continues as to how these implications or effects may be measured to account for health disparities over the span of a human lifetime, or even generations (45). In terms of health status, morbidity and mortality, current epidemiologic research indicates a persistent disparity between ethnic minority populations and ethnic majority populations (45). Ethnic discrimination is increasingly recognized as a determinant of ethnic health inequalities; growing evidence suggests pathways linking perceived discrimination and negative health outcomes (7). In general, it is hypothesized that ethnic discrimination is causally related to health status and ethnic disparities through physiological responses to chronic psychosocial stress, in turn resulting in elevated morbidity and mortality rates (6,46).

The idea that a person's experiences over a lifetime can have a cumulative effect on their health is a central idea within social epidemiology. The study of long-term effects of social exposures (such as discrimination) during childhood, adolescence, young adulthood and later adult life on the risk of chronic disease has been defined as a life course approach to chronic disease epidemiology (47). Such studies include biological, behavioural and psychosocial pathways that operate over an individual's life course, as well as across generations, to influence the course of chronic disease (48). Ethnic discrimination is thought to affect health through a number of pathways: (1) limited access to social resources such as employment, education and/or increased exposure to risk factors (such as criminal behaviours); (2) negative affective/cognitive and other pathopsychological processes; (3) allostatic load and other pathophysiological processes; (4) reduced engagement with healthy behaviours (such as exercise) and/or increased adoption of unhealthy behaviours (such as substance abuse: alcohol, drugs and/or other medication) either directly as stress coping or indirectly via reduced self-regulation; and (5) direct physical injury caused by ethnic-based violence (49).

There is substantial evidence that discrimination is related to symptoms of depression and anxiety (5,6,18), both of which have negative implications for physical health (3). Self-reported general health (SRH) is one of the most commonly examined physical health outcomes. SRH is of special interest because of its strong association with other physical health indicators, such as diabetes, obesity and coronary heart disease (CHD) (3,50).

This study is a continuation of a former study and attempts to broaden the investigation into self-reported ethnic discrimination – and associations with a number of health measures – in the indigenous (Sami) population. In our previous studies, we found ethnic discrimination to be associated with poorer self-reported physical and mental health outcomes (18). In combination with socioeconomic inequalities, the discrimination appeared to account for the health divergence between Sami and non-Sami populations of rural municipalities (including the city of Alta) of Central and North Norway.

In the first step of this work, we did not explore the association between discrimination and physical health indicators, such as CVD, chronic muscle pain, diabetes, obesity and metabolic syndrome. Therefore, in this study we are particularly interested in exploring links between discrimination and the physical health outcomes mentioned. Thus, we evaluate each of these indicators of health status in our empirical analysis.

## Objective

The objective of this study is to examine associations between self-reported ethnic discrimination and health outcomes in the rural Sami population of Central and North Norway.

## Methods

### Survey design

The SAMINOR survey provided data on the health and living conditions of mixed adult populations (containing both Sami and non-Sami peoples). The data were collected in 2003 and 2004. Invitations to participate in the survey were sent to residents of the given municipalities registered in the National Registry (In Norwegian: *Folkeregisteret*). A total of 27,151 people in the age bracket 36–79 were invited; 16,538 responded (yielding a participation rate of 60.9%). A large proportion of the population was considered to be living in rural areas. The Regional Committee for Medical and Health Research Ethics of North Norway approved the study; participants provided written, informed consent. The questionnaires were self-administered and available in both the Norwegian and Sami languages. Participants reporting a minimum of 1 Sami identity mark, and responding to questions regarding ethnic discrimination and health outcomes, were included in the analysis (sample size: n=4,389). Further details on the collection process and methods have been published previously by Lund et al. (51).

### Key variables

#### Ethnicity

It is difficult to accurately determine the ethnic makeup of North Norway as the information on ethnicity in public records remains insufficient. Due to assimilation policies, many Sami have abandoned their Sami identity and avoid reporting Sami ethnicity. The Sami Act of 1987 (52) combines self-identification and linguistic requirements in order for a person to be defined as “Sami” according to the law. As described in §2–6 of the Act, a person may be included in the Sami electoral register if the individual provides a self-declaration of Sami identity (a “subjective” criterion) *and* that the person has or at least 1 parent, grandparent or great-grandparent using or having used Sami as a language at home, or that the person is the child of a person already enrolled in the Sami census (an “objective” criterion) (52). The SAMINOR questionnaire included questions regarding the language spoken at home, the language spoken by respondents’ parents and the language spoken by respondents’ grandparents. The available responses were *Sami*, *Norwegian*, *Kven* or *Other (specify)*. Questions regarding the ethnic background of respondents and respondents’ parents were supplied with the same response options. Respondents were also asked about self-perceived ethnicity. For all the above questions, respondents were allowed to supply more than 1 answer. Based on responses to these questions, participants were included in the study if they reported at least 1 Sami identifier (i.e. *Sami language spoken by the respondent*, *Sami background*, or *Self-perceived Sami ethnicity*). However, this is a broad definition of Sami ethnicity. In the sample, 78.9% of the Sami persons had at least 2 Sami-speaking grandparents; 34.0% of the Sami persons had maternal and paternal grandparents, both parents and themselves speaking the Sami language at home. The variables are described in detail by Lund et al. (51), and Hansen et al. (17,19,20).

#### Ethnic discrimination

Participants were asked: “Have you ever experienced discrimination or harassment on account of your ethnic background (Sami, Kven, Russian, Tamil, Norwegian, etc.)?” The available responses were “Very often,” “Sometimes,” “Rarely” and “Never.” The question does not restrict such experiences to a certain time interval in the respondent's life and is therefore a measure of lifetime experience. We dichotomized this variable into *Exposed* (exposed to discrimination “Very often” or “Sometimes”) and *Unexposed* (“Rarely” or “Never” exposed to discrimination). This single-item assessment of ethnic discrimination has previously been presented in several articles (17,19,20).

The discrimination measure was further stratified into Sami majority and Sami minority areas. The Sami population was considered to be in majority in the municipalities of Kautokeino, Karasjok, Nesseby, Tana and Porsanger. These municipalities make up part of the *Sami Language Administrative District*, within which individuals are granted the right to use the Sami language in certain contexts (53). Sami minority areas were identified by the municipalities of Lebesby, Kvalsund, Loppa, Alta, Kvænangen, Kåfjord, Storfjord, Lyngen, Lavangen, Skånland, Evenes, Tysfjord, Narvik (Vassdalen) Hattfjelldalen, Grane (Majavatn), Røyrvik, Namsskogen (Trones and Furuly), Snåsa (Vinje) and Røros. The Sami majority areas were characterized by having a Sami majority population in general (Kautokeino, Karasjok, Nesseby and Tana) or in certain areas of the municipality (Porsanger) and long-time proponents of Sami language, culture and primary industries (including reindeer husbandry). Tana, Nesseby and Porsanger also have a large coastal Sami population, but these coastal areas are distinguishable from the other coastal Sami municipalities as they have a relatively large proportion of individuals reporting Sami ethnicity (54).

Combining the 2 factors created 4 different categories in terms of discrimination and location: *Unexposed in majority area*, *exposed in majority area*, *unexposed in minority area* and *exposed in minority area*.

#### Self-reported health variables

Three types of CVD were measured using the question: “Have you ever had … myocardial infarction (heart attack) (Yes/No), angina pectoris (heart cramp) (Yes/No) or cerebral stroke/brain haemorrhage (Yes/No).” Missing values were considered negative responses.

Two questions were used to measure family history of CVD: “Do you have any relatives who have, or have ever had, any of the following conditions? Please note the age at which they were diagnosed with the illness” and “Myocardial infarction before the age of 60” or “Cerebral stroke.” The available choices were: “Mother,” “Father,” “Sister,” “Brother,” “Children” and “No one.”

Participants were asked about chronic muscle pain: “In the past 12 months, have you suffered from stiffness/pain in muscles/joints for a duration of at least 3 months? (Yes/No).”

#### Laboratory analyses and physical examinations

Waist circumference (WC) was measured midway between the lower margins of the ribs and iliac crest with the individual standing and breathing normally. The cut-off points of WC were selected to identify health risks associated with excess abdominal fat according to the World Health Organization (WHO) definition (55). Central/abdominal obesity was defined as WC≥102 cm in men and WC≥88 cm in women. The methods used and procedures followed for the measurement of blood pressure, total cholesterol, HDL cholesterol, triglycerides and glucose are described in detail elsewhere (35). All blood samples in the SAMINOR study were non-fasting. We used self-reported diabetes and information about anti-diabetic medication to define diabetes. In addition, non-fasting blood glucose level ≥11.1 was defined as having diabetes (56). The WHO defines metabolic syndrome as the presence of central obesity (WC≥102 cm in males and WC≥88 cm in females) plus any 2 of 4 additional factors: Elevated triglyceride levels >1.7 mmol/l, low HDL cholesterol levels <1.03 mmol/l in males and <1.29 mmol/l in females, elevated blood pressure (systolic BP≥130 mm Hg or diastolic BP≥0.85 mmHG) and non-fasting blood glucose level >11.1 (56).

### Other variables

The logistic regression models were adjusted (entered simultaneously) for age, gender, ethnicity, marital status, education, alcohol consumption, smoking and physical activity.

Participants’ education level was measured using the question: “How many years of schooling/education have you completed?”

Self-reported marital status was measured as single, married or de facto relationship (cohabitants).

Smoking was based on the question: *Are you currently, or were you previously a daily smoker:* “Yes, currently,” “Yes, previously” or “Never”?

Alcohol intake was measured using the question: “In the past 12 months, how often have you consumed alcohol?” The available responses ranged from “I have never consumed alcohol” to “Four to seven times per week.”

Level of physical activity was measured using the question: “Describe your exercise and physical exertion in your spare time.” Four response options were available from “Reading, watching TV, or other sedentary activity” to “Participation in intensive exercise or sports competitions regularly (several times a week).”

### Data analysis

IBM SPSS Statistics Version 22 and Stata Version 13 for Mac were used to conduct statistical analyses. Fisher's exact chi-square tests were applied to compare exposed and unexposed groups (Tables I and II). The age-adjusted prevalence rates presented in Tables III and IV were based on logistic regression estimates (using the ADJPROP module (57) in Stata Version 13). Logistic regression was used to evaluate the associations between ethnic discrimination in Sami majority/minority areas and health outcomes/risk factors (Table V). All 5 models were adjusted (entered simultaneously) for age, gender, ethnicity, marital status, education level, alcohol consumption, smoking status and physical activity level.

**Table I:** Characteristics of the males study sample by minority/majority area and discriminated against status^a^

	Exposed (n=254)		Unexposed (n=923)		P^b^

	%	n	%	n	
Minority					
Mean age (µ, sd)	54.6	(10.5)	55.8	(11.4)	0.04^c^
Marital status (married)	57.7	145	61.4	567	0.21
Education≥13 years	30.3	73	27.2	237	0.34
Alcohol conception (≥1 times/week)	25.6	64	29.2	260	0.26
Never smoking	28.9	72	25.3	232	0.48
Physical activity≤1 hour per week	24.1	54	20.4	166	0.27
Self-perceived Sami ethnicity	68.5	172	28.9	261	<0.001
Sami speaking at home	46.1	89	13.8	132	<0.001
Majority	Exposed (n=290)		Unexposed (n=682)		
Mean age (µ, sd)	53.2	(10.8)	54.2	(11.1)	0.59^c^
Marital status	53.8	156	53.2	363	0.87
Education≥13 years	26.0	73	25.5	167	0.86
Alcohol conception (≥1 times/week)	22.4	64	25.4	170	0.32
Never smoking	22.9	66	27.9	188	0.15
Physical activity≤1 hour per week	29.3	79	24.8	158	0.19
Self-perceived Sami ethnicity	85.4	246	69.1	462	<0.001
Sami speaking at home	83.9	234	63.0	424	<0.001

a *Exposed* (exposed to discrimination “Very often” or “Sometimes”) and *Unexposed* (“Rarely” or “Never” exposed to discrimination).

b p Value from Likelihood Ratio Fisher's Exact Test for difference between Unexposed and Exposed groups.

c Two-sample t-test with unequal variances.

Subgroups may not be total due to missing values.

**Table II:** Characteristics of the females study sample by minority/majority area and discriminated against status^a^

	Exposed (n=195)		Unexposed (n=966)		P^b^

	%	n	%	n	
Minority					
Mean age (µ, sd)	53.6	(11.0)	53.7	(11.7)	0.80^c^
Marital status (married)	57.9	113	62.0	599	0.29
Education≥13 years	37.4	67	30.5	277	0.05
Alcohol conception (≥1 times/week)	10.3	19	17.5	163	0.01
Never smoking	34.0	66	34.6	333	0.88
Physical activity≤1 hour per week	24.0	40	19.8	169	0.23
Self-perceived Sami ethnicity	70.3	137	27.4	256	<0.001
Sami speaking at home	46.1	89	13.8	132	<0.001
Majority	Exposed (n=286)		Unexposed (n=793)		
Mean age (µ, sd)	52.2	(10.8)	52.8	(11.2)	0.20^c^
Marital status (married)	56.3	161	55.0	436	0.70
Education≥13 years	42.3	112	36.7	274	0.07
Alcohol conception (≥1 times/week)	12.6	35	12.9	100	0.28
Never smoking	38.2	109	39.3	309	0.64
Physical activity≤1 hour per week	22.8	60	26.1	193	0.29
Self-perceived Sami ethnicity	91.4	254	75.6	589	<0.001
Sami speaking at home	83.9	234	67.7	531	<0.001

a *Exposed* (exposed to discrimination “Very often” or “Sometimes”) and *Unexposed* (“Rarely” or “Never” exposed to discrimination).

b p Value from Likelihood Ratio Fisher's Exact Test for difference between Unexposed and Exposed groups.

c Two-sample t-test with unequal variances.

Subgroups might not be total due to missing values.

**Table III:** Age-specific and total prevalence rates self-reported health outcomes/risk factors in males by majority/minority area and discrimination status

	Minority area					Majority area				
	
	Discriminated					Discriminated				
	
	Against		not			Against		not		
	
	n	%	n	%	P^a^	n	%	n	%	P^a^
Cardiovascular disease										
36–49 years	3	3.8	4	1.5	0.22	4	3.5	8	3.3	0.92
50–59 years	18	21.2	32	11.0	**0.02**	12	13.2	22	10.4	0.48
60–79 years	23	31.5	102	30.9	0.92	24	31.2	55	26.4	0.43
Total crude	44	18.5	138	15.6	0.29	40	14.2	85	12.8	0.58
Total age-adjusted^b^	44	15.1	138	10.6	**0.05**	40	11.3	85	9.2	0.29
Chronic muscle pain										
36–49 years	28	34.1	83	31.7	0.68	33	30.0	73	30.8	0.88
50–59 years	44	51.6	120	40.7	0.20	47	50.5	79	38.0	0.42
60–79 years	32	44.4	122	37.9	0.30	32	43.8	66	32.2	0.07
Total crude	104	42.4	325	37.0	0.12	112	40.6	218	33.5	**0.04**
Total age-adjusted^b^	104	42.3	325	36.5	0.09	112	40.5	218	33.3	**0.04**
Diabetes										
36–49 years	5	6.2	5	1.9	**0.04**	2	1.8	3	1.2	0.70
50–59 years	9	10.7	16	5.5	0.09	7	7.8	13	6.1	0.60
60–79 years	15	20.8	31	9.4	**0.01**	5	6.4	17	8.3	0.61
Total crude	29	12.2	52	5.9	**0.001**	14	5.0	33	5.0	0.98
Total age-adjusted^b^	29	11.1	52	4.8	**0.001**	14	4.5	33	4.3	0.91
Obesity										
36–49 years	14	17.3	38	14.2	0.50	21	18.4	29	11.9	0.10
50–59 years	18	19.6	43	14.7	0.26	19	21.1	32	15.4	0.23
60–79 years	17	23.3	65	19.9	0.51	17	23.0	26	12.6	**0.03**
Total crude	49	19.9	146	16.5	0.20	57	20.5	87	13.2	**0.005**
Total age-adjusted^b^	49	19.8	146	15.9	0.17	57	20.3	87	13.2	**0.006**
Metabolic syndrome										
36–49 years	7	9.7	9	3.9	**0.05**	6	6.1	9	4.1	0.45
50–59 years	4	5.4	14	5.6	0.96	9	11.4	15	8.1	0.39
60–79 years	6	10.3	23	8.3	0.61	4	6.8	8	4.4	0.47
Total crude	17	8.3	46	6.0	0.24	19	8.0	32	5.5	0.17
Total age-adjusted^b^	17	8.2	46	5.6	0.2	19	7.9	32	5.4	0.18

a p Values from likelihood ratio tests for difference between discriminated against and not discriminated against groups. p Values in bold indicate significant difference between discriminated against and not discriminated against groups equal or higher than<0.05 level.

b The age-adjusted probabilities are based on logistic regression estimates.

**Table IV:** Age-specific and total prevalence rates self-reported health outcomes/risk factors in females by majority/minority area and discrimination status

	Minority area					Majority area				
	
	Discriminated					Discriminated				
	
	Against		not			Against		not		
	
	n	%	n	%	P^a^	n	%	n	%	P^a^
Cardiovascular disease										
36–49 years	2	3.0	2	0.6	0.06	1	0.9	2	0.6	0.81
50–59 years	8	13.3	22	8.4	0.24	6	6.7	14	5.9	0.80
60–79 years	16	30.8	71	24.5	0.34	10	15.6	39	18.4	0.61
Total crude	26	14.5	95	10.5	0.12	17	6.3	55	7.2	0.61
Total age-adjusted^b^	26	8.2	95	5.9	0.18	17	3.4	55	3.7	0.80
Chronic muscle pain										
36–49 years	42	57.5	175	49.9	0.23	49	41.5	109	36.1	0.30
50–59 years	29	50.9	148	56.1	0.48	45	50.6	107	45.3	0.40
60–79 years	35	71.4	142	50.2	**0.01**	28	47.5	85	41.1	0.38
Total crude	106	59.2	465	51.8	0.07	122	45.9	301	40.4	0.12
Total age-adjusted^b^	106	57.8	465	51.3	0.11	122	46.5	301	40.3	0.07
Diabetes										
36–49 years	3	4.3	6	1.7	0.17	4	3.4	10	3.2	0.90
50–59 years	6	10.2	16	6.1	0.26	6	6.7	11	4.7	0.46
60–79 years	9	18.4	26	9.3	0.06	1	1.6	23	10.8	**0.02**
Total crude	18	10.2	48	5.4	**0.02**	11	4.1	44	5.8	0.29
Total age-adjusted^b^	18	8.9	48	4.9	**0.04**	11	3.6	44	5.0	0.32
Obesity										
36–49 years	28	38.4	94	27.4	0.06	27	23.1	87	28.4	0.27
50–59 years	29	50.0	102	38.3	0.10	36	40.4	97	41.3	0.89
60–79 years	25	48.1	150	49.5	0.85	37	61.7	117	56.0	0.43
Total crude	82	44.8	346	37.9	0.08	100	37.6	301	40.1	0.47
Total age-adjusted^b^	82	45.5	346	37.6	**0.05**	100	37.6	301	39.8	0.54
Metabolic syndrome										
36–49 years	12	21.4	30	10.9	**0.03**	8	8.2	25	10.3	0.57
50–59 years	7	21.2	30	15.6	0.43	10	16.4	34	20.4	0.50
60–79 years	11	28.9	54	27.3	0.83	10	30.3	45	33.3	0.74
Total crude	30	23.6	114	17.1	0.08	28	14.7	104	19.1	0.17
Total age-adjusted^b^	30	23.3	114	16.2	**0.05**	28	14.2	104	17.2	0.32

a p Values from likelihood ratio tests for difference between discriminated against and not discriminated against groups. p Values in bold indicate significant difference between discriminated against and not discriminated against groups equal or higher than<0.05 level.

b The age-adjusted probabilities are based on logistic regression estimates.

**Table V:** The association (Odd ratio with 95% confidence intervals) between self-reported ethnic discrimination^a^ “ever” and health outcomes/risk factors: the SAMINOR study

Self-reported ethnic discrimination	CVD^b^		Chronic muscle pain^c^		Diabetes^d^		Obesity^e^		Metabolic syndrome^f^	

	OR	(95% CI)	OR	(95% CI)	OR	(95% CI)	OR	(95% CI)	OR	(95% CI)
Discriminated against										
Exposed minority	2.30	(1.49–3.55)	1.68	(1.31–2.14)	2.42	(1.55–3.76)	1.58	(1.20–2.09)	1.55	(1.02–2.35)
Unexposed minority	1.42	(1.02–1.98)	1.27	(1.07–1.50)	0.94	(0.64–1.36)	0.96	(0.79–1.17)	0.82	(0.61–1.10)
Exposed majority	1.04	(0.65–1.64)	1.30	(1.04–1.62)	1.02	(0.62–1.68)	1.34	(1.04–1.72)	1.26	(0.85–1.87)
Unexposed majority	1		1		1		1		1	

a All models were adjusted for age, gender, ethnicity (self-perceived Sami ethnicity), marital status, education level, alcohol consumption, smoking status and physical activity level.

b Lifetime cardiovascular disease was measured by 3 questions: “Do you have, or have you had: Myocardial infarction (heart attack)?”, “Angina pectoris (heart cramp)?” or “Cerebral stroke/brain haemorrhage?”. The estimate is also adjusted for cardiovascular disease in family.

c Question asked: “Have you during the last year suffered from pain and/or stiffness in muscles or joints that has lasted for at least 3 months?”.

d Diabetes were measured by questions about self-reported diabetes type 2, information about anti-diabetic medications, and non-fasting blood glucose level>11.1.

e Waist circumference (WC)≥102 cm in males and WC≥88 cm in females.

f Metabolic syndrome (MetS) is defined by World Health Organization's definition.

## Results

The characteristics of the study sample are presented in Table I (2,149 males) and Table II (2,240 females). A total of 1,025 respondents (males and females; 23.4% of the study sample) reported having experienced discrimination. Males reporting experience of discrimination were more likely to report self-perceived Sami ethnicity, and were more likely to use the Sami language at home. Among females, significant differences between exposed and unexposed in the distribution of education ≥13 years, alcohol consumption, self-perceived Sami ethnicity and use of Sami language at home were observed. Age was negatively correlated with experienced discrimination (i.e. younger participants more frequently reported having experienced discrimination) (data not shown).

Tables III and IV show the age-specific and total prevalence rates of CVD, chronic muscle pain, diabetes, obesity and metabolic syndrome. Sami males in minority areas reporting self-perceived discrimination show higher rates of CVD, diabetes and metabolic syndrome. Males living in Sami majority areas reporting exposure to discrimination are more likely to be obese and suffer from chronic muscle pain (in comparison to those unexposed to discrimination). Sami females exposed to discrimination living in a minority area show elevated rates in the occurrence of diabetes, obesity and metabolic syndrome (in comparison to females unexposed to discrimination living in the same area). However, in the case of Sami females living in Sami majority areas there were no significant differences in terms of health outcomes between exposed and unexposed groups. For Sami females in general, CVD appeared to be unassociated with experienced ethnic discrimination.

Table V presents 5 adjusted odds ratio models for the association between ethnic discrimination and CVD, chronic muscle pain, diabetes, obesity and metabolic syndrome (adjusted for age, gender, ethnicity, marital status, education level, alcohol consumption, smoking status and physical activity level).

Self-reported experience of ethnic discrimination was associated with all negative health outcomes in adjusted models for Sami living in minority areas. The association tended to be stronger for CVD and diabetes. However, to be female was a protective factor for CVD (data not shown). Self-reported ethnic discrimination had an impact on WC and chronic muscle pain in Sami populations (in both majority and minority areas) when compared to Sami majority populations unexposed to discrimination.

## Discussion

This study continues the task of monitoring ethnic discrimination as a health determinant in Norway's Sami population (17,19,20). We found exposure to ethnic discrimination to be associated with a range of negative health outcomes. The findings support existing evidence of the negative health impacts of ethnic discrimination for the indigenous (Sami) people of Norway. In the case of Sami populations living in minority areas, significant associations were established between exposure to ethnic discrimination and a number of health indicators: CVD, chronic muscular pain, diabetes, obesity and metabolic syndrome.

About 1 in 4 Sami persons report experiencing discrimination, according to our previous study (17). The previous study operated with a 3-tiered Sami ethnicity variable, within which Sami individuals with the strongest Sami affiliation reported more ethnic discrimination (36.0%) and those with the weakest Sami affiliation reported less (12.3%) (17). This article, however, operates with a non-stratified ethnicity variable, i.e. a generalized Sami ethnicity variable (applying an “average” of the above figures). Studies conducted among indigenous Maori in New Zealand (46,58) Australian Aboriginal (59) and young Sami in Sweden (60) reported prevalence of discrimination level similar to our study.

Previous studies have found associations between racism (defined as self-reported experiences of racial or ethnic discrimination) and a range of adverse health outcomes (5,16,61). Review articles have focused on adverse mental and physical health outcomes. Racism was found to be closely associated with both negative mental health and health-related behaviours in a review by Paradies (5). However, for physical health outcomes, consistent evidence for a correlation with racism has proven elusive (62). The present study contributes evidence for a relationship between discrimination and physical health outcomes (such as obesity and diabetes), potentially revealing the pathway linking ethnicity and cardiovascular risk.

The association between exposure to ethnic discrimination and self-reported CVD in minority areas is consistent with our previous study, in which (for Sami males) a strong correlation was found between marginalization and CVD (54). However, we did not find any significant difference between exposed and unexposed Sami females regarding CVDs. This was consistent with the previous study (54).

In our study we found discrimination to be associated with several chronic conditions, such as chronic muscle pain, diabetes and metabolic syndrome. Only a small number of studies have examined ethnic or cultural differences in muscle pain and the association with psychosocial factors (38), such as discrimination. Studies conducted in Canada and Australia show that indigenous peoples are at greater risk of musculoskeletal pain than the general population (63,64). One study of adult Sami subjects revealed that differences between Sami and non-Sami populations are not dependent upon known socioeconomic factors, suggesting that there are other factors that may explain the observed differences (37). Metabolic syndrome is established as a precursor state to CVD and is linked to increased risk of type 2 diabetes (48). However, the link between metabolic syndrome and psychosocial factors is less understood. Central obesity as a component of metabolic syndrome likely correlates with socioeconomic factors. This corresponds with our previous study, in which we found ethnic discrimination to be associated with poorer self-reported physical health (19). Those findings are consistent with a recent study, in which everyday discrimination was revealed to be associated with a greater count of chronic conditions (65). Also, in a recent meta-analysis, Pasco and Smart Richmann (7) found that self-reported discrimination had a significant effect on physical health outcomes, including general self-reported health, diseases and chronic physical conditions, such as diabetes and CVD.

The pathway leading from discrimination to health are certain to be complex and multidimensional (45). The general susceptibility hypothesis (66) states that social factors (discrimination, for example) affect health by creating vulnerability (susceptibility) to disease in general and not to any specific disorder. The hypothesis stems from the observation of many social conditions being linked to a wide range of diseases. Although specific diseases are influenced by behavioural, environmental, biological and genetic factors, socially stressful conditions may interact with these factors and contribute to illness and early mortality (48). Ethnic discrimination can affect health through a number of pathways (62). Discrimination may act as a chronic stressor with wide ranging health impacts (6,67). We can understand the experience of discrimination as a stressor that broadly impacts the somatic health of an individual. The cumulative experience of stress may impact on a variety of chronic and infectious diseases through neuroendocrine-mediated biological pathways (48). Such experiences may contribute to the ethnic divide in terms of the health of the Sami and non-Sami populations of Norway (19). The finding applies to indigenous peoples worldwide (6). Proponents of the biopsychosocial model of discrimination (45) argue that heightened and prolonged psychological and physiological responses to experiences of discrimination can negatively affect the physical health of an individual; the cognitive, emotional and biological cost associated with constant adjustments to stressors potentially leads to allostatic load (68). Everyday experience of discrimination may elicit acute physiological activation, including altered heart rate and blood pressure, and the release of stress hormones such as cortisol (69). Prolonged response to stress triggers pathogenic mechanisms, which over time confers elevated risk for cardiovascular, neuroendocrine and immunologic stress-related diseases (70).

In the modern nation-state and in the international community, the Sami people have progressed from being strongly stigmatized to being treated as equals. However, despite the revitalization and integration of Sami culture, language and identity, the Sami people still report ethnic discrimination more frequently than their peers in the general population (18). In some areas (predominantly Sami areas), “Saminess” is a given; in others (predominantly Ethnic Norwegian areas), one must actively struggle for a visible Sami presence to be accepted (71). In the latter areas there is less structural and practical support for Sami culture, language and identity (19). These factors may explain why we find such a strong association between experienced ethnic discrimination and several chronic conditions in Sami populations living in minority areas and a weaker association between discrimination and health outcomes in the Sami populations of majority areas: the stronger Sami civil society in the majority area may have a protective effect in regards to stress exposure (19,54).

A great number of studies conducted across the world have revealed robust associations between perceived discrimination and poor somatic health outcomes in ethnic minorities/indigenous peoples, even when controlled for a number of confounders, such as socioeconomic status, lifestyle factors and other demographic variables (7,58,72). This study contributes specifically to the literature on Sami health and living conditions, and, more generally, to the growing literature on discrimination and somatic health. In summary, addressing and reducing the ethnic discrimination faced by the Sami people may prove to be a viable avenue for the promotion of good health.

## Strengths and weaknesses

The relatively large sample means that the findings of this study are representative for the Sami population between 36 and 79 years of age living in semi-rural areas of North Norway. The study contributes empirical evidence to the understanding of the relationship between ethnic discrimination and multiple health outcomes.

However, some limitations need to be noted, such as the fact that the study (51) has a cross-sectional design. Cross-sectional studies are limited in terms of the temporal ordering of variables and causal inference. Several longitudinal studies suggest that the association between discrimination and physical illness appears after a significant latency period (62,73), suggesting that cross-sectional studies are biased towards type II errors in relation to physical outcomes (62). Therefore, causality cannot be taken for granted.

In our study, exposure to ethnic discrimination is defined as life course experienced discrimination. Some of the outcome variables indicate former or current status (CVD, diabetes or obesity); other health variables are measured as being within the time frame of the past 12 months (chronic muscular pain). The different time frames used when measuring exposure to discrimination and outcome variables may have biased our estimates of the association between ethnic discrimination and health.

Using single-item measurement in the assessment of life course experienced ethnic discrimination has some limitations; the extent to which the current single-item question under- or over-estimate actual levels of discrimination in Sami population is unknown.

Our measure of ethnic discrimination examines whether self-reported experience of discrimination is associated with specified health outcomes; it does not measure implicit discrimination (for example, differences in health care treatment) or institutional, population level discrimination. In the future we will seek to use instruments (several items) to measure everyday experience of ethnic discrimination in different domains (such as school, work, medical care setting, public setting, etc.).

Other problems that have been identified with the reliability and validity of the measurement of life course discrimination and the association with multiple health outcomes include: unreliability of recall, recall bias, criterion validity and construct validity (74). Disease status was self-reported, not medically confirmed. The reported accuracy of self-reported diagnoses is inconsistent in the literature. However, given that previous studies conducted in North Norway used self-reporting in comparable populations, we believe that our estimates are valid; our goal was to identify individuals who have experienced CVD, chronic muscle pain or diabetes (28).

Participants were included in the study if they reported at least 1 Sami identifier (i.e. *Sami language spoken by the respondent*, *Sami background*, or *Self-perceived Sami ethnicity*). The application of alternative definitions of ethnicity could affect risk estimates (53). We are aware of the fact that the definition of ethnicity has its limitations. In Norway, (as well as in Sweden and Finland), Sami ethnicity is primarily defined using self-identification. Proficiency in the Sami language is a secondary determinant of Sami ethnicity that typically does not require “direct” language skills; so-called retroactive language skills suffice (52). Consequently, our logistic regression analyses were controlled for self-perceived Sami ethnicity.

We have little information about non-respondents, other than that they tend to be young, single males. With a participation rate of 61%, selection bias is a possibility. The differences between respondents and non-respondents are often important but rarely significant enough to undermine studies (75).

## Conclusion

The findings of this study highlight the need to acknowledge and address ethnic discrimination as an important determinant of health and wellbeing for the indigenous (Sami) people of Norway.
